# Self-complementary adeno-associated virus serotype 6 mediated knockdown of ADAMTS4 induces long-term and effective enhancement of aggrecan in degenerative human nucleus pulposus cells: A new therapeutic approach for intervertebral disc disorders

**DOI:** 10.1371/journal.pone.0172181

**Published:** 2017-02-16

**Authors:** Demissew Shenegelegn Mern, Anja Tschugg, Sebastian Hartmann, Claudius Thomé

**Affiliations:** Department of Neurosurgery, Innsbruck Medical University, Innsbruck, Austria; Wake Forest Institute for Regenerative Medicine, UNITED STATES

## Abstract

Inhibition of intervertebral disc (IVD) degeneration, which is often accompanied by painful inflammatory and immunopathological processes, is challenging. Current IVD gene therapeutic approaches are based on adenoviral gene delivery systems, which are limited by immune reactions to their viral proteins. Their applications in IVDs near to sensitive neural structure could provoke toxicity and immunological side-effects with neurological deficits. Self-complementary adeno-associated virus (scAAV) vectors, which do not express any viral gene and are not linked with any known disease in humans, are attractive therapeutic gene delivery vectors in degenerative IVDs. However, scAAV-based silencing of catabolic or inflammatory factor has not yet been investigated in human IVD cells. Therefore, we used scAAV6, the most suitable serotype for transduction of human nucleus pulposus (NP) cells, to knockdown the major catabolic gene (ADAMTS4) of IVD degeneration. IVD degeneration grades were determined by preoperative magnetic resonance imaging. Lumbar NP tissues of degeneration grade III were removed from 12 patients by nucleotomy. NP cells were isolated and cultured with low-glucose. Titre of recombinant scAAV6 vectors targeting ADAMTS4, transduction efficiencies, transduction units, cell viabilities and expression levels of target genes were analysed using quantitative PCR, fluorescence microscopy, fluorescence-activated cell sorting, 3-(4,5-dimethylthiazolyl-2)-2,5-diphenyltetrazolium bromide assays, quantitative reverse transcription PCR, western blot and enzyme-linked immunosorbent assays during 48 days of post-transduction. Transduction efficiencies between 98.2% and 37.4% and transduction units between 611 and 245 TU/cell were verified during 48 days of post-transduction (p<0.001). scAAV6-mediated knockdown of ADAMTS4 with maximum 87.7% and minimum 40.1% was confirmed on day 8 and 48 with enhanced the level of aggrecan 48.5% and 30.2% respectively (p<0.001). scAAV6-mediated knockdown of ADAMTS4 showed no impact on cell viability and expression levels of other inflammatory catabolic proteins. Thus, our results are promising and may help to design long-term and less immunogenic gene therapeutic approaches in IVD disorders, which usually need prolonged therapeutic period between weeks and months.

## Introduction

Intervertebral discs (IVDs) have a particular load-bearing organization that distributes loading consistently on the vertebral bodies, withstands spinal compression and provides flexibility in the spine. IVDs consist of hydrated nucleus pulposus (NP), radially aligned annulus fibrosus (AF) and cartilaginous endplates (EP) [1–2]. NP contains predominantly proteoglycans, hyaluronic acid and type II collagen. The major proteoglycan constituent is aggrecan, which is connected by link protein to the hyaluronic acid. A fibril network of AF made from various types of collagen and glycoproteins enfolds the NP [1–2]. Painful IVD degeneration is associated with structural failure of IVD tissues, which is often accompanied by inflammatory and immunopathological processes [3–5].

Degenerative NP cells have been shown to induce inflammatory cytokines such as interleukin-1β (IL-1β) and tumor necrosis factor-α (TNF-α), which evidently induce overexpression of the catabolic factor ADAMTS4 (A disintegrin and metalloproteinase with thrombospondin motifs 4) [6–8]. Overexpression of ADAMTS4 has been shown to adversely affect the biomechanics of IVD [6–8]. ADAMTS4 (aggrecanase-1) and ADAMTS5 (aggrecanase-2) are identified to be the primary degrading agents of aggrecan in the gene family of metalloproteinases. Thus far, 20 genetically different members of ADAMTSs have been identified in humans [9–16]. While ADAMTS5 is constitutively expressed in IVD cells, overexpression of ADAMTS4 is induced by the inflammatory cytokines such as IL-1β and TNF-α [6, 16–17]. Moreover, in degenerative IVDs and articular cartilages the levels of ADAMTS4 were shown to increase with grades of degeneration [7–8, 17]. Although TIMP-3 (tissue inhibitor of metalloproteinases-3) and fibronectin are described to be the physiological inhibitors of ADAMTS4, their expression levels seem to be inadequate for effective inhibition of ADAMTS4 activity [18–20, 7–8]. Therefore, progressive overexpression of ADAMTS4 seems to be a key therapeutic target in degenerative joint and IVD diseases.

Current gene therapeutic approaches used to target degenerative IVD cells *in vitro* or in small animal models are based mostly on adenoviral gene delivery systems [21–26]. Although adenoviral vectors can be used for high level and persistent expression of therapeutic genes, their therapeutic potential is limited by the immune reactions to their viral proteins. Accordingly, their applications in spinal discs near to sensitive neural structure could provoke toxicity and immunological side-effects that could result in neurological deficits and serious pain [27–31].

Adeno-associated viruses (AAVs), which do not express any viral gene and are not linked with any known disease in humans, have become attractive therapeutic gene delivery vectors [32–35]. The advantage of self-complementary AAV (scAAV) vectors over standard AAV vectors is based on their ability to fold upon themselves and immediately form transcriptionally competent double-stranded DNA, which allow them to bypass the limiting aspects of second-strand synthesis and shorten the lag time before transgene expression; and that could increase their biological efficiency [36].They can thus preferably be used than standard AAV vectors, especially for cloning of small therapeutic molecules. Therefore, in our preliminary study we aimed at identifying the optimal scAAV vectors for effective and long-term transduction of human NP cells. By screening different scAAV serotypes, we identified scAAV6 as the most suitable serotype for effective and long-term transduction of human NP cells [37]. However, functional applications of recombinant scAAV vectors in human IVD research have not yet been examined. Thus, we determined to investigate a functional application of recombinant scAAV6 vectors in degenerative human NP cells by inducing shRNA mediated knockdown of ADAMTS4. scAAV6 mediated knockdown of ADAMTS4 showed long-term enhancement of aggrecan without any impact on cell viability and the expression of other inflammatory, catabolic or matrix proteins. Thus, this study may help to establish less immunogenic gene therapeutic approaches in degenerative IVD disorders.

## Materials and methods

### Recruitment of NP tissues from patients

**Fig 1:**
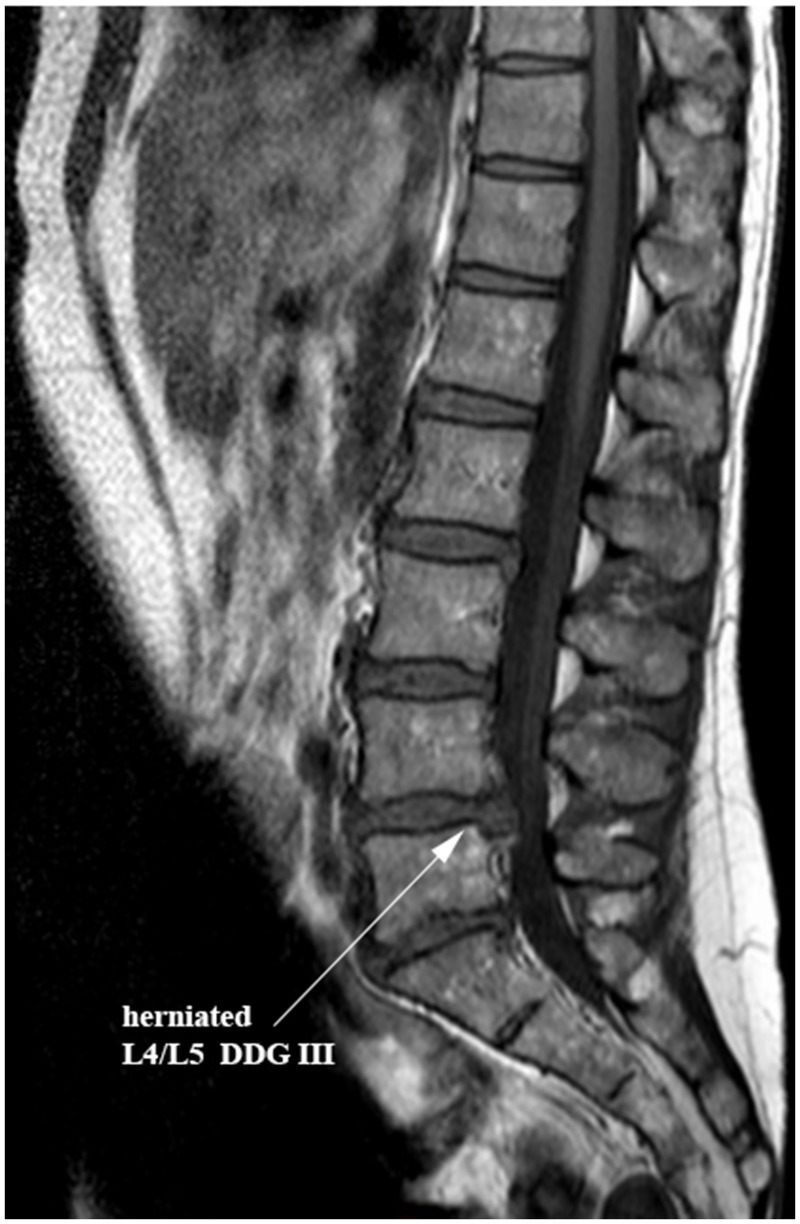
A representative MRI picture of the IVD samples. 12 patients with lumbar discs of degeneration grade III (mean age of 49 years: range 34–61 years) were involved in this study. A representative MRI exhibits a herniated lumbar disc (L4/L5) of degeneration grade III (DDG III) with nerve root compression that remained unresponsive to non-operative treatment for six weeks.

The local research ethics committee (Innsbruck Medical University: project AN2014-0027 333/4.24) authorized this experimental study of human IVD tissues. Samples of NP tissues were recruited from patients during lumbar disc surgery with informed consents of the patients. Patients provided their written informed consent to participate in this study. Inclusion criteria for surgery were intervertebral disc herniation with nerve root compression detected on MRI, which correlated to primary symptoms that remained unresponsive to non-operative treatment for six weeks, or demonstrated progressive neurological deterioration in the face of conservative treatment. 12 lumbar discs of Pfirman degeneration grade III from 12 patients (mean age of 49 years: range 34–61 years) were involved in this study (Table 1). Disc degeneration grade (DDG) was determined by preoperative MRI [38–39]. A representative MRI picture of the 12 IVD samples, which presents a herniated lumbar disc of DDG III with nerve root compression, is shown in Fig 1. Lumbar NP tissues were recruited from NP compartment by nucleotomy and immediately taken to the laboratory in sterile phosphate buffered saline solution (PBS) (Sigma-Aldrich) for direct isolation of NP cells.

**Table 1:** Samples of lumbar NP tissues recruited from 12 patients.

Sample	Disc level	DDG	Age/Sex
1	L4/L5	III	34/F
2	L4/L5	III	40/F
3	L5/S1	III	41/M
4	L4/L5	III	45/F
5	L4/L5	III	47/M
6	L5/S1	III	48/M
7	L5/S1	III	50/M
8	L4/L5	III	54/F
9	L4/L5	III	55/M
10	L5/S1	III	55/F
11	L4/L5	III	58/M
12	L5/S1	III	61/F

### Isolation and monolayer expansion of NP cells

IVD tissues isolated from patients were immediately brought to the laboratory and washed twice in PBS (1000 x g, 2 min). NP tissues were separated from AF tissues on the basis of their macroscopic morphology (identification of the innermost lamellar rings of the AF) and finely minced into small fragments of approximately 2 mm^3^. NP tissues were then digested with 0.02% w/v pronase (Sigma-Aldrich) (37°C, 5% CO2, 1 h) in 20 ml DMEM (Dulbecco's Modified Eagle's Medium), which contained 1% penicillin/streptomycin, 1% glucose and 10% FCS (Fetal Calf Serum) (Sigma-Aldrich). After filtration of samples through sterile 75 gm nylon mesh filters (Sigma-Aldrich) and centrifugation of the supernatants (1000 x g, 5 min), pellets were resuspended in 20 ml culture medium and digested with 0.02% w/v collagenase II (Sigma-Aldrich) (37°C, 5% CO2, 3 h). Samples were again filtered and centrifuged as stated above. Pellets were then resuspended in 5 ml culture medium and cultured in 25 cm^2^ tissue culture flask till reaching 100% confluent by changing the culture medium every two days (37°C, 5% CO2, about 2 weeks). For later experiments NP cells were cryopreserved at -196°C in culture medium containing 30% FCS and 15% dimethyl sulfoxide (DMSO) (Sigma-Aldrich).

### Plasmid construction

Construction of the shuttle plasmid containing the human cytomegalovirus (CMV) promoter and U6 promoter (a polymerase III promoter), which encodes the expression cassettes of *emerald green fluorescent protein* (emGFP) and shRNA, was based on the scAAV shuttle plasmid described before [40–41]. The plasmids encoding the shRNA that targets the 3'-terminal region of ADAMTS4 or the non-target control shRNA are named as pAAV-ADAMTS4 and pAAV-Ctrl respectively. The respective shRNA sequences were cloned between *Bam*HI and *Hind*III restriction sites of the shuttle plasmid. The sense sequences of the shRNA targeting ADAMTS4 and the control shRNA are listed below respectively:

5´-GCCAAGCGCTTTGCTTCACTGTTCAAGAGACAGTGAAGCAAAGCGCTTGGCTTTTT-3´

5´- CATCTTACCGAGCATGACGTTCAAGAGACGTCATGCTCGGTAAGATGTTTTT-3‘

The helper plasmid of AAV6 (pDP6rs) was purchased from PlasmidFactory GmbH & Co.KG (Bielefeld, Germany).

### Production and purification of the recombinant scAAV6 vectors

For the production of the recombinant scAAV6 vectors human embryonic kidney 293 (HEK293) cells were grown in DMEM, which contained 1% penicillin/streptomycin, 4.5% glucose and 10% FCS, and passaged 2 times prior to transfection. For the transfection 5 x 10^6^ HEK293 cells were cultured in 15 cm culture dish with 20 ml culture medium till reaching 70–80% confluence. To prepare the transfection medium 30 μg of shuttle plasmid pAAV-ADAMTS4 or pAAV-Ctrl were mixed with 96 μg of the helper plasmid (pDP6rs). The respective mixture was added to 2.5 ml of 300 mM calcium phosphate (Sigma-Aldrich) and gently mixed with 2.5 ml of 2 x HBS (Hepes Buffered Saline) (Sigma-Aldrich). The transfection medium was directly pipetted to the culture dish and changed with DMEM containing 2% FCS after 6 hours of incubation (37°C, 5% CO2). After 72 h of transfection culture medium was collected and cells were harvested by trypsinization and combined for centrifugation (2000 x g, 5 min). Pellet was resuspended in a serum-free DMEM (2.5 ml) and subjected to eight rounds of freeze/thaw cycles by alternating the tube between dry ice-ethanol bath and 37°C water bath. AAV supernatant was then collected by centrifugation (8000 x g, 30 min) and stored at -80°C for subsequent purification. Purification of the recombinant scAAV6 vectors was performed as described before [42]. Briefly, the recombinant scAAV6 vectors were purified from freeze/thaw-supernatants by iodixanol (Sigma-Aldrich) gradient centrifugation and iodixanol was removed by running the iodixanol fractions through PD10 gel filtration columns (GE Healthcare). 10 fractions of 1 ml eluate were collected and fractions 4 to 6 were pooled for quantification. The recombinant scAAV6 vectors (AAV6-ADAMTS4 or AAV6-Ctrl) were then quantified by using quantitative PCR.

### Titration of the recombinant scAAV6 vectors with quantitative PCR

For quantitative PCR (qPCR) of the recombinant scAAV6 vectors the LightCycler 480 (Roche Applied Science) and the TaqMan Gene Expression Master Mix (Life Technologies) were applied. 1× master mix, supplemented with 200 nM sense, 200 nM antisense primers of 5´-ITR, 250 nM ITR-probe and 2 μl of the template DNA, was used for PCR reactions in 20 μl of final volume. The sequences of the applied ITR primers and ITR-probe are listed below:

5´-ITR-sense: GGAACCCCTAGTGATGGAGTT

5´-ITR-antisense: CGGCCTCAGTGAGCGAG

ITR-probe: 6FAM-CACTCCCTCTCTGCGCGCTCG-BHQ1.

Standard, negative control and samples were run in three replicates of a 96 well-plate. The genomic DNA of the shuttle plasmid was used as a standard as described before [41]. The PCR program had an initial denaturation step at 95°C for 10 min, 40 cycles of denaturation at 95°C for 15 s, an extension at 60°C for 1 min and a melt curve stage (65°C to 95°C, increment 0.5°C). Data analysis was performed using the Applied Biosystems StepOne software v2.1 (Life Technologies). Three independent qPCRs were performed for each probe.

### Evaluating transduction efficiencies of the recombinant scAAV6 vectors in NP cells

NP cells were seeded in 24-well plates at a density of 1 x 10^5^ cells per well (about 50% confluent) and cultured for 24 hours in 500 μl DMEM containing 1% FCS (37°C, 5% CO2). Cells were then transduced with the recombinant scAAV6 vectors (AAV6-Ctrl or AAV6-ADAMTS4) at a dose of 5000 vector genome copy per seeded cell (5000 vg/c). Transduction efficiencies were evaluated every 2 days for the first 16 days and weekly up to 48 days by using fluorescence microscopy (AxioVert.A, Carl Zeiss). Transduction efficiencies were then quantified by using flow cytometry-assisted cell sorting (FACS) on day 8, 16, 24, 32 and 48 of post-transduction. For FACS analyses 1 x 10^5^ cells per sample were counted using MoFlo cell sorter (Beckman Coulter). The number of GFP-positive cells was quantified according to the manufacturer’s protocol. Briefly, a MoFlo cell sorter with a 100-mm flow cell tip and a flow rate of 12000 events per second, an extension wavelength of 488 nm and a laser power of 110 W was applied. Three independent FACS analyses with duplicate samples were performed for each probe.

### Titration of transduction units in NP cells

For titration of transduction units (the number of recombinant scAAV6 vectors internalized into NP cells) NP cells were seeded, transduced with AAV6-Ctrl or AAV6-ADAMTS4 and harvested on day 2, 8, 16, 24, 32 and 48 as described above. In order to remove the recombinant scAAV6 vectors that remained attached to the surface of NP cells, pellets were washed three times with PBS. Pellets were then resuspended in a final volume of 100 μl and subjected to eight rounds of freeze/thaw cycles by alternating the tube between the dry ice-ethanol bath and the 37°C water bath. After centrifugation (17000 x g, 5 min) supernatants were used for titration of the transduction units (TU). The qPCR with the 5´-ITR primers and ITR-probe was used as described before. The shuttle vector was used as standard in 10-fold dilutions from 10^6^–10^3^ copies/μl. Non-transduced NP cells were used as a background control. Data analysis was performed using the Applied Biosystems StepOne software v2.1 (Life Technologies). Three independent qPCRs with triplicate samples were performed for each probe.

### NP cell viability assay

To analyse the effects of the recombinant scAAV6 vectors on NP cell viability, the number of viable cells were quantified on day 2, 8, 16, 24, 32 and 48 of post-transduction by using 3-(4, 5-dimethylthiazolyl-2)-2,5-diphenyltetrazolium bromide assay (MTT Assay Kit, Molecular Probes). NP cells were seeded, transduced with AAV6-Ctrl or AAV6-ADAMTS4 and harvested as defined above. Non-transduced NP cells were used as control. Pellets were washed twice with PBS, resuspended in 250 μl culture medium and duplicates of 100 μl were plated into flat-bottomed 96-well plate. Control wells of medium alone were added to provide the blanks for absorbance readings. After incubation for recovering (37°C, 5% CO_2_, 24 h), 10 μl MTT reagent was added to each well and incubated for 3 h. Then 100 μl of the SDS-HCl solution was added to each well and further incubated for 4 h. A microtiter plate reader Infinite 200 (TECAN) was used to measure the absorbance at 570 nm. The average value of the blank duplicate readings was subtracted from the average values of the sample duplicate readings and the number of viable cells was calculated from the standard curve. Three independent MTT assays with duplicate samples were performed for each probe.

### Culture of NP cells on collagen I scaffold

A sterile scaffold of 24-well plate format made from bovine collagen type I (Viscofan Bioengineering) was used for culturing of NP cells. The scaffold was taken out of the blister with sterile forceps and placed into the well that was filled with 250 μl PBS (pH 7.3 without Ca^2+^ / Mg^2+^). After 20 min of incubation at room temperature, the scaffold was attached to the bottom of the well and the remaining PBS was removed. The plate was left overnight in the operating laminar flow hood and the next day the scaffold was equilibrated with 250 ml pre-warmed culture medium (37°C, 5% CO_2_, 10 min). Then 1 x 10^5^ monolayer transduced NP cells in 250 ml culture medium were pipetted onto the pre-wetted scaffold that was placed into the 24-well plate. Non-transduced NP cells were used as control. Similar to non-transduced NP cells transduced NP cells showed almost 100% cell adherence to the scaffold. Cell culture on the scaffold was performed (37°C, 5% CO2) by changing the culture medium every two days. On day 8, 16, 24, 32 and 48 NP cells were harvested by digestion of the scaffold with 0.02% w/v collagenase (Sigma-Aldrich) in 250 μl medium (37°C, 5% CO_2_, 3 h). Cell suspension was filtered through sterile 75 gm nylon mesh filter and pelleted by centrifugation (1000 x g, 5 min). Cell pellet was washed twice in 1 ml PBS (1000 x g, 5 min) and processed for expressional analyses.

### Quantitative reverse transcription PCR

To examine the effects of AAV6-ADAMTS4 on the mRNA levels of ADAMTS4 and aggrecan, quantitative reverse transcription PCR (RT-qPCR) was used. 1 x 10^5^ NP cells were seeded, transduced and cultured on the scaffold as defined before. NP cells cultured on the scaffold were harvested on day 8, 16, 24, 32 and 48 and total RNA was isolated by using the RNeasy Plus Mini Kit (Qiagen). DNA contamination was removed by DNase 1 (Sigma-Aldrich) and quantification of total RNA at 260 nm was performed using Biospectrometer (Eppendorf). Equal amounts of RNA were used for reverse transcription (RT) and cDNAs were synthesized using TaqMan Reverse Transcription Reagents (Applied Biosystems). The mRNA levels of ADAMTS4 and aggrecan were measured by qPCR using TaqMan gene expression assays and LightCycler 480 as described before. Beta-actin was used as internal standard. Non-transduced and AAV6-Ctrl transduced NP cells were used as control. Three independent RT-qPCRs with triplicate samples were performed for each probe. The data of the relative mRNA levels were numerically presented using the comparative 2^-ΔΔCT^ method.

The sequences (5'-3') of the primers and probes used for RT-qPCR are given below:

ADAMTS4-sense: AACATGCTGCACGACAACAG

ADAMTS4-antisense: GTAGCCGTTGTCCAGGAAGT

ADAMTS4-probe: 6FAB-AGCACCAGCAGGCACGTGAT-BHQ1

Aggrecan-sense: AGGGACACCAACGAGACCTA

Aggrecan-antisense: GGAAGGTGAACTTCTCGGGG

Aggrecan-probe: 6FAB-GTACTGCTTCGCCGAGGAGA-BHQ1

Beta-actin-sense: CAGAAGGACAGCTACGTGGG

Beta-actin-antisense: CATGTCGTCCCAGTTGGTCA

Beta-actin-probe: 6FAB- GACCCTGAAGTACCCCATCG-BHQ1

### Western blot

To verify the impacts of AAV6-ADAMTS4 on the protein expression levels of ADAMTS4 and aggrecan western blot was applied. 1 x 10^5^ NP cells were seeded, transduced, cultured on the scaffold and harvested on day 8 and 48 as described before. For total protein isolation harvested cells were lysed for 20 min by using ice cold radio-immunoprecipitation assay (RIPA) buffer (Sigma-Aldrich), which was supplemented with protease and phosphatase inhibitors (Sigma-Aldrich). Samples were centrifuged for 10 min (14000 × g, 4°C) and supernatants were used for western blot analysis. Total protein concentration in samples and controls was determined by BCA Protein Assay Kit (Thermo Scientific). Equal amounts of proteins were separated by sodium dodecyl sulfate polyacrylamide gel electrophoresis (SDS-PAGE, Sigma-Aldrich) and transferred to polyvinylidene fluoride (PVDF) membranes (Merck Millipore). Anti-ADAMTA4 antibody (SAB1411668, Sigma-Aldrich) and anti-aggrecan antibody (SAB4500662, Sigma-Aldrich) were used as primary antibodies. Interactions between antigens and primary antibodies were detected on the membrane by using horseradish peroxidase-conjugated goat anti-rabbit secondary antibody (A0545, Sigma-Aldrich) and Amersham ECL Western Blotting Detection kit (GE Healthcare Life Sciences). Image J software was used to quantify protein bands.

### Enzyme-linked immunosorbent assay

To examine the presence of unspecific effects of AAV6-ADAMTS4 on the expression levels of other intervertebral disc relevant proteins, the enzyme-linked immunosorbent assay (ELISA) was used. 1 x 10^5^ NP cells were seeded, transduced, cultured on the scaffold and harvested on day 8, 16, 24, 32 and 48 as described before. After total protein isolation and quantification, 100 μg of total protein extracts from each sample were used for ELISA. In addition to ADAMTS4 and aggrecan the protein expression levels of ADAMTS5, IL-1β (interleukin-1β), TNF-α (tumour necrosis factor alpha) and collagen II were quantified using ELISA kits according to the instruction manuals (R&D Systems, Uscn Life Science Inc). A microplate reader Infinite 200 (TECAN) was used to measure the absorbance at 450 nm with wavelength correction set to 540 nm. The average values of the blank duplicate readings were subtracted from the average values of the sample duplicate readings and protein concentrations were calculated from the standard curve. Three independent assays with duplicate samples were performed for each probe.

### Statistical data analysis

Landis and Koch based interpretations with κ statistics and agreement percentage among two observers were used to estimate the reliability of MRI evaluations [38–39]. The software IBM SPSS Statistics 22 (Armonk New York USA) was used for statistical data analyses. 1-way ANOVA and pairwise comparisons using Bonferroni post hoc test were applied to compare the data of treated and untreated samples as well as the data at different time points. Significance was set at p < 0.05.

## Results

### Reliability of MRI grading

The MRI rating of IVD degeneration grade (DDG) between two observers (the interobserver reliability agreement) was calculated using agreement percentage and kappa (κ) statistics [38–39]. The agreement was rated as excellent with 100% frequency of agreement and κ = 1.00.

### Determination of recombinant scAAV6 genome copies by qPCR

Recombinant scAAV6 vectors AAV6-ADAMTS4 (encoding the shRNA targeting ADAMTS4) and AAV6-Ctrl (encoding non-target control shRNA) were produced, purified and quantified as described in the methods. For both vectors high final titer between 5.7 x 10^11^ and 2.1 x 10^12^ copies were determined from 3 x 10^7^ HEK293 cells.

### Evaluation of transduction efficiencies using fluorescence microscopy and FACS

**Fig 2:**
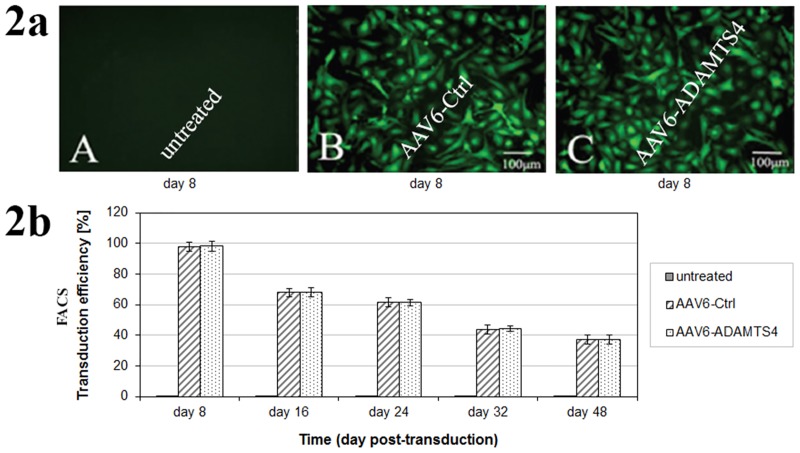
Transduction efficiencies of the recombinant scAAV6 vectors in NP cells. 1 x 10^5^ NP cells were seeded and transduced with 5000 vg/c GFP packing recombinant scAAV6 vectors: AAV6-Ctrl (encoding non-target control shRNA) or AAV6-ADAMTS4 (encoding the shRNA targeting ADAMTS4). For both vectors similar transduction efficiencies were determined by fluorescence microscopy and FACS (Fig 2a & 2b). Fluorescence micrographs were acquired every 2 days for the first 16 days and weekly up to 48 days and the highest transduction efficiencies were recorded on day 8 of post-transduction (Fig 2a): A) untreated NP cells, B) NP cells treated with AAV6-Ctrl and C) NP cells treated with AAV6-ADAMTS4. For quantitative evaluation of the transduction efficiencies, GFP positive NP cells were quantified by FACS on day 8, 16, 24, 32 and 48 (Fig 2b). Data represent the mean with standard deviation (SD) of three independent experiments, each performed in triplicate. Error bars indicate SD values.

In fluorescence microscopy both AAV6-ADAMTS4 and AAV6-Ctrl consistently showed similar transduction efficiencies and their highest transduction efficiencies were recorded on day 8 of post-transduction (Fig 2a). Moreover, FACS consistently detected equivalent numbers of GFP positive cells for both vectors (p ≥ 0.648). As in fluorescence microscopy, the highest transduction efficiencies were detected on day 8 with an average of 98.0% and 98.2% GFP positive cells for AAV6-Ctrl and AAV6- ADAMTS4 respectively. However, declining transduction efficiencies were detected in the course of 48 days. The recorded mean transduction efficiencies on day 48 were 37.2% for AAV6-Ctrl and 37.4% for AAV6-ADAMTS4 respectively (Fig 2b). Age and gender did not show any additional effect on the transduction efficiencies.

### Evaluation of transduction units and NP cell viability

**Fig 3:**
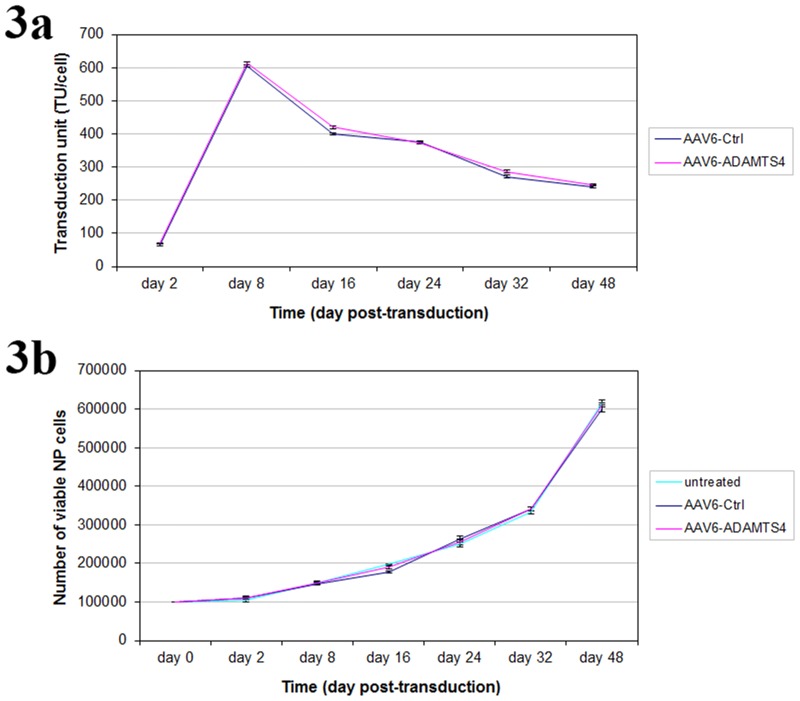
Transduction units and cell viabilities of the recombinant scAAV6 vectors. 1 x 10^5^ NP cells were transduced with recombinant scAAV6 vectors AAV6-Ctrl or AAV6-ADAMTS4 at a viral dose of 5000 vg/c. NP cells were harvested on day 2, 8, 16, 24, 32 and 48 of post-transduction to determine the number of transduction units by qPCR and cell viabilities by MTT assay. Comparable numbers of transduction units per cell (TU/cell) were determined in AAV6-Ctrl or AAV6-ADAMTS4 treated NP cells (Fig 3a). Equivalent numbers of viable cells were determined in AAV6-Ctrl or AAV6-ADAMTS4 treated and untreated NP cells (Fig 3b). Data represent the mean values with standard deviation (SD) of three independent experiments, each performed in triplicate. Error bars indicate SD values.

As only a portion of AAV6-ADAMTS4 or AAV6-Ctrl are able to enter NP cells, quantifying the number of internalized recombinant vectors (transduction units: TU) is very important to obtain reproducible functional results. The qPCR results showed that both vectors consistently reached similar numbers of TU per cell (TU/cell) (p ≥ 0.216) and the TU reached a plateau within the first 8 days of post-transduction. On day 8 the maximum mean transduction units were (609 ± 4.45) and (611 ± 5.56) TU/cell for AAV6-Ctrl and AAV6-ADAMTS4 respectively. After day 8 the number of transduction units continuously decreased and on day 48 they reached the mean values of (242 ± 4.92) TU/cell for AAV6-Ctrl and (245 ± 2.05) TU/cell for AAV6-ADAMTS4 (Fig 3a). To examine the potential effect of AAV6-Ctrl and AAV6-ADAMTS4 on the viability of NP cell, MTT assays were performed on day 2, 8, 16, 24, 32 and 48 of post-transduction. Similar viabilities of NP cells were confirmed after transduction with AAV6-Ctrl or AAV6-ADAMTS4. The viabilities of transduced cells were equivalent to that of the untreated cells (p ≥ 0.351) (Fig 3b). No morphological changes were observed between treated and untreated NP cells (data not shown). Age and gender did not show any additional effect on cell viability and the number of transduction units.

### Knockdown of ADAMTS4 and enhancement of aggrecan level in degenerative NP cells

**Fig 4:**
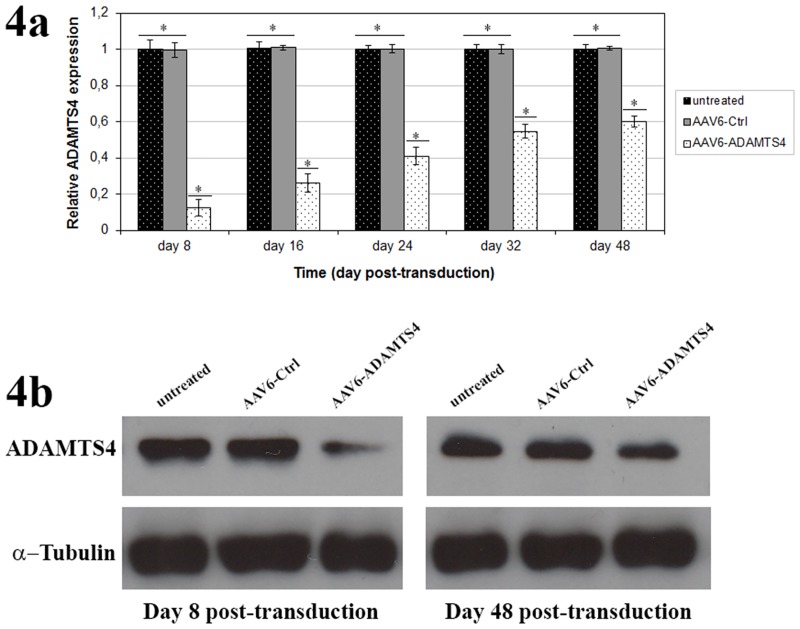
Knockdown of ADAMTS4 by scAAV6 mediated shRNA. 1 x 10^5^ NP cells were transduced with 5000 vg/c of recombinant scAAV6 vectors AAV6-Ctrl or AAV6-ADAMTS4. On day 3 of post-transduction monolayer transduced and non-transduced NP cells were transferred to scaffold and harvested on day 8, 16, 24, 32 and 48. RT-qPCR (Fig 4a) and western blot (Fig 4b) were performed to determine the Knockdown effect of AAV6-ADAMTS4 at mRNA and protein levels. The treatment of NP cells with AAV6-ADAMTS4 showed effective and long-term knockdown of ADAMTS4. The level of ADAMTS4 in AAV6-Ctrl treated and untreated cells remained unaffected. Data represent the mean values with standard deviation (SD) of three independent experiments, each performed in triplicate (*, p < 0.001).

**Fig 5:**
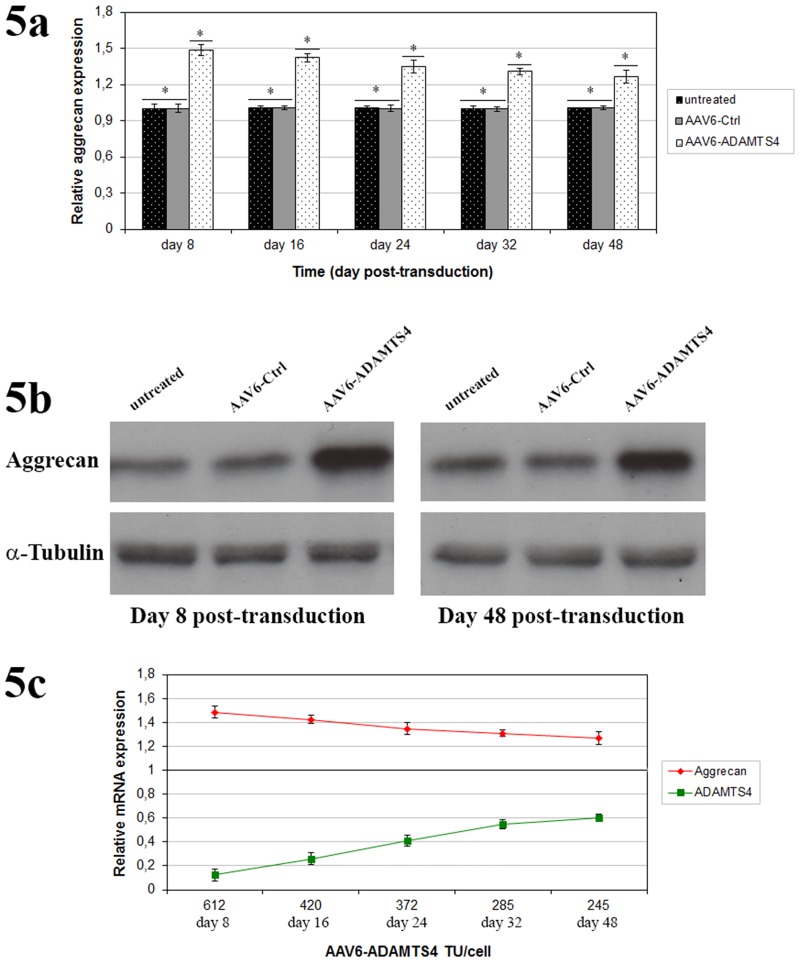
Enhancement of aggrecan induced by scAAV6 mediated knockdown of ADAMTS4. Viral dose of 5000 vg/c from recombinant scAAV6 vectors AAV6-Ctrl or AAV6-ADAMTS4 were used for transduction of 1 x 10^5^ NP cells. On day 3 of post-transduction, treated and untreated NP cells were transferred to scaffold and harvested on day 8, 16, 24, 32 and 48. The Enhancement of aggrecan level induced by ADAMTS4 Knockdown was controlled by RT-qPCR (Fig 5a) and western blot (Fig 5b). AAV6-ADAMTS4 mediated knockdown of ADMATS4 induced enhancement of aggrecan at mRNA and protein levels. Moreover, plotting the mRNA levels of ADMATS4 and aggrecan against the number of transduction units per cell (TU/cell) showed a direct correlation between the TU/cell, ADAMTS4 knockdown and enhancement of aggrecan level (Fig 5c). Data represent the mean values with standard deviation (SD) of three independent experiments, each performed in triplicate (*, p < 0.001).

In order to examine the functional application of recombinant scAAV6 vectors in degenerative NP cells, scAAV6 mediated knockdown of ADAMTS4 was attempted using the AAV6-ADAMTS4 vectors that encode shRNA directed against ADAMTS4. Using RT-qPCR the maximum knockdown of ADAMTS4 was confirmed on day 8 with an average reduction level of 87.7%. Although the knockdown effect was continuously weakened in the course of 48 days, the mean level of ADAMTS4 was still reduced by 74% on day 16 and by 40.1% on day 48 (p < 0.001) (Fig 4a). Furthermore, using western blot effective and long-term knockdown of ADAMTS4 was confirmed at protein level on day 8 and 48 of post-transduction (Fig 4b). On day 8 the ADAMTS4 knockdown could increase the level of aggrecan at an average of 48.5%. As the knockdown effect was continuously weakened in the course of time, the enhancement of aggrecan level was similarly declined. However, the level of aggrecan was still improved by 42.3% on day 16 and by 30.2% on day 48 (p < 0.001) (Fig 5a). Similarly, effective and long-term enhancement of aggrecan was confirmed by western blots on day 8 and 48 (Fig 5b). The treatment of NP cells with AAV6-Ctrl had no effect on the levels of ADAMTS4 and aggrecan. Their expression levels in AAV6-Ctrl treated and untreated NP cells were similar (Figs 4a & 5a) (p ≥ 0.652). Furthermore, plotting the mRNA levels of ADAMTS4 and aggrecan against the number of TU per cell (TU/cell) demonstrated a direct correlation between TU, ADAMTS4 knockdown and enhancement of aggrecan (Fig 5c). Age and gender did not show any additional effect on ADAMTS4 knockdown.

### Specificity of AAV6-ADAMTS4 mediated knockdown

**Fig 6:**
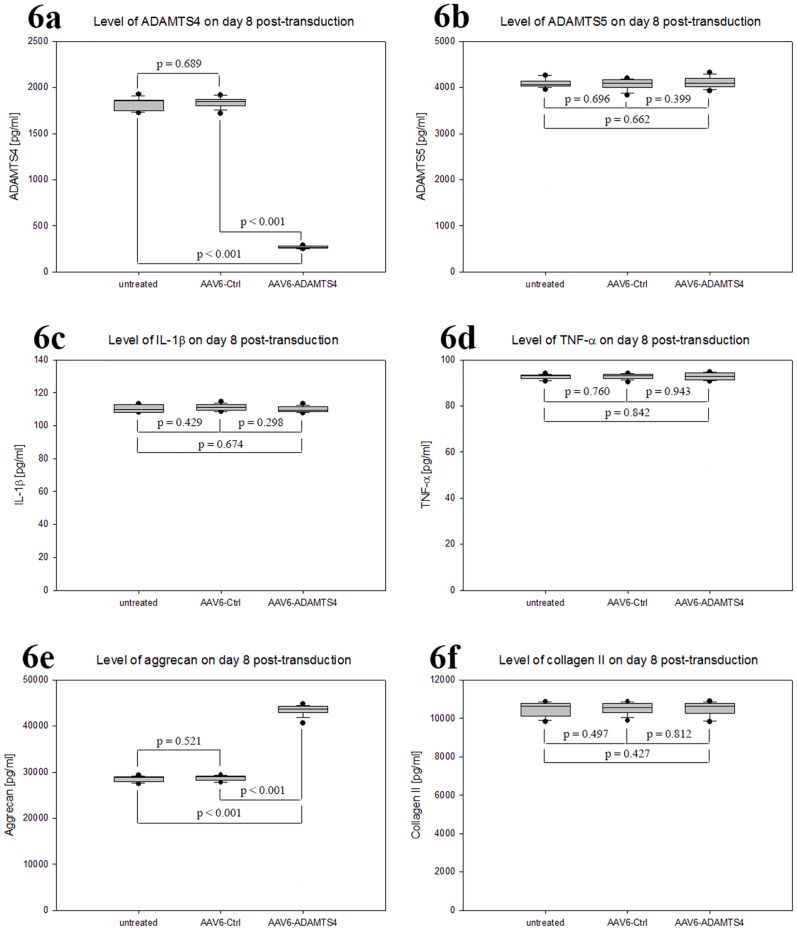
Specific impact AAV6-ADAMTS4 on the levels of ADAMTS4 and aggrecan. To examine whether AAV6-ADAMTS4 induces unspecific changes in the expression levels of catabolic, inflammatory and matrix proteins in degenerative NP cells, the expression levels of ADAMTS5, IL-1β, TNF-α and collagen II were examined by using ELISA. 1 x 10^5^ NP cells were seeded, transduced with 5000 vg/c of AAV6-Ctrl or AAV6-ADAMTS4 and transferred to scaffold on day 3 of post-transduction. On day 8, 16, 24, 32, 48 NP cells were harvested from scaffold and 100 μg of total protein extracts were used for ELISA. Box plots with whiskers represent the expression levels on day 8 (Fig 6a–6f). AAV6-ADAMTS4 mediated knockdown showed specific impact on the levels of ADAMTS4 and aggrecan (Fig 6a & 6e). The expression levels of ADAMTS5, IL-1β, TNF-α and collagen II were not affected (Fig 6b, 6c, 6d & 6f). In AAV6-Ctrl treated and untreated cells the levels of all tested proteins remained unaffected (Fig 6a–6f). Data represent the mean values of three independent experiments, each performed in duplicate (p < 0.05).

Treatment of NP cells with AAV6-ADAMTS4 vectors, encoding the shRNA directed against ADAMTS4, showed high transduction efficiencies, efficient transduction units and no impact on the viability of NP cells. Moreover, AAV6-ADAMTS4 promoted effective and long-term knockdown of ADAMTS4 that resulted in increased level of aggrecan. To prove whether AAV6-ADAMTS4 induces unspecific changes in the expression levels of vital catabolic, inflammatory and matrix proteins in NP cells, the expression levels of ADAMTS5, IL-1β, TNF-α and collagen II were examined by using ELISA. The results of ELISA on day 8 showed that the average level of ADAMTS4 was reduced in AAV6-ADAMTS4 treated cells by 85.6% compared to that in AAV6-Ctrl treated and untreated cells. Their recorded mean expression values in pg/ml were (265 ± 12.5), (1843 ± 47.8) and (1830 ± 66.4) respectively (p < 0.001) (Fig 6a). At the same time the level of aggrecan was enhanced in AAV6-ADAMTS4 treated cells by 52.6% compared to that in AAV6-Ctrl treated and untreated NP cells. Their mean expression values in pg/ml were (43472 ± 1092), (28696 ± 642) and (28487 ± 604) respectively (p < 0.001) (Fig 6e). Conversely, the expression levels ADAMTS5, IL-1β, TNF-α and collagen II were not affected in AAV6-ADAMTR4 treated cells. Their expression levels were similar to that in AAV6-Ctrl treated and untreated NP cells (Fig 6b, 6c, 6d & 6f). In the course of 48 days only the levels of ADAMTS4 and aggrecan were changed. On day 48 the level of ADAMTS4 in AAV6-ADAMTS4 treated cells was reduced by 46.8% with mean value of 867 ± 26 pg/ml and the level of aggrecan was enhanced by 28.3% with mean value of 36795 ± 270 pg/ml (p < 0.001). In AAV6-Ctrl treated and untreated cells the levels of ADAMTS4 and aggrecan remained unaffected. The results these experiments indicated that the treatment of NP cells with AAV6-ADAMTS4 had only specific effects on the levels of ADAMTS4 and aggrecan. Age and gender did not show any additional effect on the specificity of AAV6-ADAMTS4 mediated knockdown.

## Discussion

AAV vectors that do not express any viral protein and are not linked with any known disease in humans are important for gene therapeutic approaches in degenerative IVD diseases. Their applications in IVDs near to sensitive neural structure could minimise toxicity and immunological side-effects, which could result in neurological deficits and serious pain. Current studies regarding the potential use of AAV in therapeutic approaches of degenerative IVD disorders are only based on the use of standard AAV2 vectors [43–46]. However, various tissues have been detected to be more susceptible to other AAV serotypes than AAV2 [47–48]. Since lack of tissue tropism and pre-existing immune response are major problems of AAV-based gene therapeutic applications, we aimed in our preliminary study at identifying the optimal AAV serotype for efficient and long-term transduction of human NP cells. scAAV6 was identified as the most suitable vector for efficient and long-term transduction of human NP cells [37]. However, the functional application of scAAV vectors in human IVD research is a new approach that has not yet been examined. Therefore, we determined to perform functional experiments to prove the therapeutic potential of recombinant scAAV6 vectors in degenerative human NP cells. scAAV6 vectors were used rather than standard AAV6 vectors, as they can bypass the limiting aspects of second-strand synthesis and possibly increase their biological efficacy [35].

In spite of big advantages of using scAAV vectors, there are some limitations. Because of their small genome size (4.5 kbp) scAAV vectors are not convenient for cloning of large genes. Possibilities are currently being explored to override this limiting capacity. For example the ITRs of two AAV genomes can anneal to form head to tail concatemer that could almost double the coding capacity of the vector. However, scAAV vectors have become very interesting for the delivery of small therapeutic genes (up to 3.0 kbp) and shRNAs in RNAi approaches [49].

For the application of the RNA interference (RNAi) technique, we generated the recombinant scAAV6 vectors encoding the shRNA targeting ADAMTS4 (AAV6-ADAMTS4) or the non-target control shRNA (AAV6-Ctrl). ADAMTS4 is considered as a key therapeutic target, since it is the main degrading agent of aggrecan and its level continuously rises in degenerating NP cells [13–15, 6–8]. In this study NP cells of disc degeneration grade III (Table 1 & Fig 1) transduced with the recombinant scAAV6 vectors AAV6-ADAMTS4 or AAV6-Ctrl showed high and similar long-term transduction efficiencies in the course of 48 days (p ≥ 0.648). The maximum transduction efficiencies were confirmed on day 8 (Fig 2a & 2b) and the transduction efficiencies of both vectors were steadily weakened between day 8 and 48. They were declined from 98.2% on day 8 to 37.4% on day 48 for AAV6-ADAMTS4 and from 98.0% on day 8 to 37.2% on day 48 for AAV6-Ctrl (Fig 2b). Similarly, the number of transduction units per cell (TU/cell) reached their maximum values on day 8 and their values also monotonically decreased between day 8 and 48 from 611 to 245 TU/cell for AAV6-ADAMTS4 and from 609 to 242 TU/cell for AAV6-Ctrl (p < 0.001) (Fig 3a). Both vectors did not show any impact on the viability or proliferation rate of NP cells (Fig 3b). These results are consistent with the data of our prior publication [37], which indicate that the cloned shRNA sequences had no impact on the transduction efficiencies, transduction units and cell viabilities. During the course of 48 days the transduction efficiencies and number of transduction units were monotonically reduced. Due to the fact that NP cells frequently divide, AAV vector genome got diluted over time in the growing cell population (Fig 3b). The dilution of the AAV vector genome was in line with our expectation, as recombinant AAV vectors could remain episomal without integration into chromosome 19 and thus they could possibly get lost during cell division [33–35]. Nonetheless, using AAV6-ADAMTS4 efficient and long-term knockdown of ADAMTS4 was achieved. The knockdown of ADAMTS4 and the enhancement of aggrecan in degenerative NP cells were verified in a period of 48 days by at mRNA and protein levels. The maximum knockdown of ADAMTS4 reached about 88% on day 8 and at the same time the level of aggrecan was enhanced by about 49%. Although the knockdown effect was continuously weakened to about 40% by day 48, it could still improve the level of aggrecan by about 31% (Figs 4a, 4b, 5a and 5b). Moreover, AAV6-ADAMTS4 mediated knockdown of ADAMTS4 and enhancement of aggrecan showed a direct correlation with the number of transduction units (Fig 5c). This confirms that frequent determination of transduction units is very important to ensure reproducible results in a long-term functional analysis of recombinant AAV vectors. Furthermore, the AAV6-ADAMTS4 mediated knockdown showed only specific effects on the levels of ADAMTS4 and aggrecan (Fig 6a & 6e). It had no effect on the expression levels of ADAMTS5, IL-1β, TNF-α and collagen II (Fig 6b, 6c, 6d & 6f). These results indicated that there were no inflammatory catabolic responses of NP cells to the recombinant vectors AAV6-ADAMTS4 or AAV6-Ctrl.

In this study we focused on the use of scAAV6 for effective and long-term inhibition of ADAMTS4, which is one of the direct and primary degrading agents of aggrecan in the gene family of metalloproteinases. The sites of aggrecan cleavage by ADAMTS4 are known; and the thrombospondin motif of ADAMTS4 is known to be critical for aggrecan substrate recognition and cleavage [13–15]. This verifies the direct link between these two genes. Regulation mechanism of ADAMTS4 in degenerative NP cells by the inflammatory cytokines (IL-1β and TNF-α) through MAPK and NF-κB signalling is reported [Ref. 6]. However, additional mechanisms might exist that may affect aggrecan degeneration.

We in fact used a narrow range of patient population to test this technology (12 patients with disc degeneration grade III). We showed in our previous studies [7–8] with a larger range of patient population (78 patients with disc degeneration grade III, IV and V) higher levels of ADAMTS4 and lower levels of aggrecan in grade IV and V discs. Certainly, before future *in vivo* application the efficiency of this technology need to be proved additionally in higher degenerated (grade IV and V) disc cells.

*In vivo* delivery of scAAV vectors could affect the transduction efficiencies due to serotype-specific interactions with the extracellular matrix. This could be a very challenging problem for achieving high transduction efficiencies *in vivo*. However, highly scAAV6-transduced monolayer cultured NP cells could be transferred into 3D culture to maintain their phenotypic character before *in vivo* application. This strategy could probably be an efficient and suitable method for generating large populations of scAAV6 modified NP cells. Such modified NP cells would be used in the future for cell-based regenerative treatment approaches in view of autologous or allogenic disc cell transplantation.

In conclusion, this is the first study to analyse a functional application of recombinant scAAV vectors in human IVD research. Recombinant scAAV6 vectors could be used in degenerative NP cells for effective and less immunogenic gene therapeutic approaches. AAV6-ADAMTS4 mediated knockdown of ADAMTS4 could substantially enhance the level of aggrecan, without inducing any toxicity or inflammatory catabolic responses. The effective ADAMTS4 silencing and enhancement of aggrecan that lasted more than six weeks without any inflammatory catabolic response are promising, as IVD disorders usually need prolonged therapeutic periods. However, intervertebral disc degeneration is as an age and genetics dependent molecular degeneration process, which can be accelerated by toxic and biomechanical factors. Collective involvement of these factors can result in structural failures that are often associated with pain. Therefore, combinatorial molecular therapies and physio-medical strategies that improve the biomechanics of the spine could be very useful for future *in vivo* applications.
